# Ischemic Stroke of the Artery of Percheron with Normal Initial MRI: A Case Report

**DOI:** 10.1155/2010/425734

**Published:** 2010-03-14

**Authors:** Guillaume Cassourret, Bertrand Prunet, Fabrice Sbardella, Julien Bordes, Olga Maurin, Henry Boret

**Affiliations:** ^1^Intensive Care Unit, Sainte-Anne Hospital, Boulevard Sainte Anne, 83000 Toulon, France; ^2^Department of Radiology, Sainte-Anne Hospital, 83000 Toulon, France

## Abstract

The artery of Percheron is a solitary trunk representing an uncommon anatomic variant that provides bilateral arterial supply to the paramedian thalami and the rostral midbrain. Occlusion of this artery results in bilateral thalamic and mesencephalic infarctions. The clinical diagnosis is difficult because the complex anatomy causes large clinical variability. We report a case of a comatose patient with normal early head-computed tomography and magnetic resonance imaging. A bilateral paramedian thalamic infarct due to an occlusion of the artery of Percheron was revealed two days later by a new head computed tomography. To our knowledge, this is the first report in the literature of a symptomatic patient presenting an acute Percheron stroke with normal early brain magnetic resonance imaging. Our case indicates that a normal initial magnetic resonance imaging cannot formally eliminate the diagnosis of acute stroke of the artery of Percheron. We discuss the causes of noncontributive brain magnetic resonance imaging at the onset of this acute Percheron stroke and the alternative diagnosis and therapy methods.

## 1. Introduction

**Figure 1:**
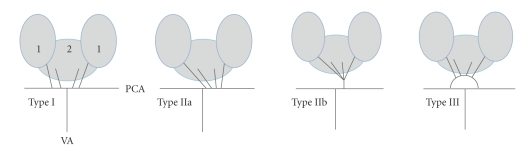
The four variations of the vascularization of the thalami and the midbrain described by Percheron (1: Thalami, 2: Midbrain, PCA: Posterior Cerebral Artery, VA: Vertebral Artery).

Percheron described four normal variations of the neurovascular anatomy of the thalami and midbrain (Figure 1). The medial part of the thalami is supplied from the posterior circulation via the perforating thalamic arteries, also called paramedian arteries [1]. Variation I is the most common, where each perforating artery arises from each left and right posterior cerebral artery. In variation IIb, the bilateral perforating thalamic arteries arise from one central artery called the artery of Percheron, which arises from the P1 segment of one posterior cerebral artery. It supplies the paramedian thalami and the rostral midbrain [2–4]. Consequently, occlusion of the artery of Percheron causes a bilateral paramedian thalamic and mesencephalic infarction [2, 5]. Magnetic resonance imaging (MRI) normally allows visualization of the initial infarct in cases of acute cerebral ischemia and is usually used in stroke centers as the primary or early secondly imaging modality. 

We report a case of a patient admitted for coma without evident cause and normal early imaging by head computed tomography (CT) and MRI. A new head CT two days later revealed a bilateral paramedian thalamic infarct at the origin of the initial symptoms.

## 2. Case Presentation

**Figure 2:**
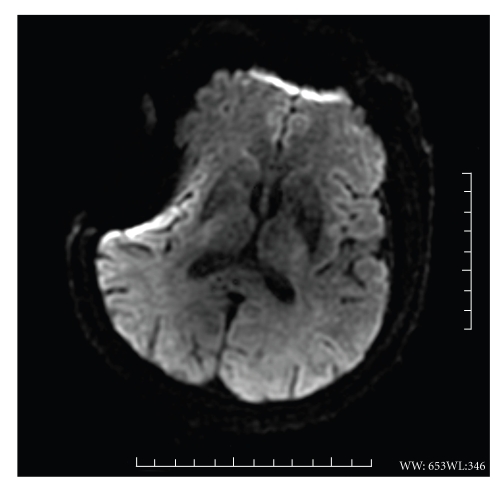
Axial cerebral DWI (b = 1000 s/mm^2^) performed 95 minutes after the onset symptoms show no ischemic lesion.

**Figure 3:**
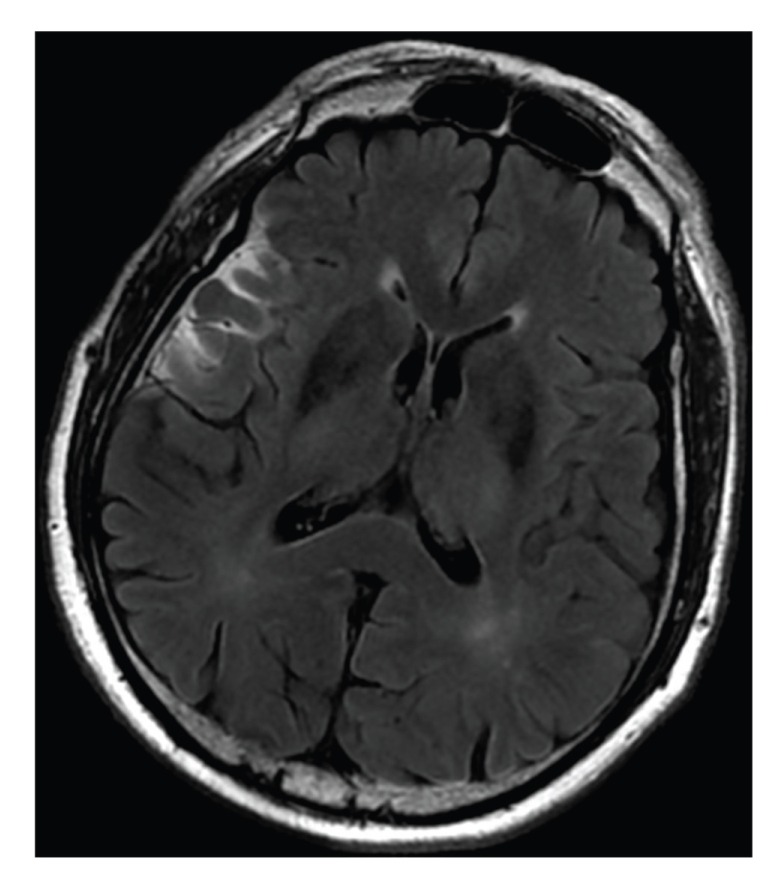
Axial cerebral FLAIR performed 95 minutes after the onset symptoms show no ischemic lesion.

A 64-year-old right-handed Caucasian man presented with a sudden loss of consciousness at home, 5 minutes after feeling discomfort with dysarthria. His past medical history included dyslipidemia, diabetes, obesity, and chronic atrial fibrillation. He was treated with long-term oral anticoagulants (fluindione), but with poor therapeutic observance. He was found comatose by the prehospital team. On examination, the patient had the following initial vital signs: temperature 36.4°C, pulse 78 beats/min, respiratory rate 16 breaths/min, and blood pressure 148/87 mmHg. On neurological examination, the Glasgow coma-scale score was 7 with localizing pain being the best motor response and no opening of eyes or verbal response. Pupillary light reflex in the right eye was 4 mm nonreactive and that in the left eye was 3 mm reactive. The deep tendon reflexes were present and symmetric. Babinski sign was found on both feet. Capillary blood glucose was found to be 123 mg/dL. The electrocardiogram showed atrial fibrillation, with no abnormal repolarization. He was intubated and mechanically ventilated and then brought to the emergency department. The initial head CT, performed 65 minutes after loss of consciousness, showed no acute hemorrhage or other abnormality. To eliminate an acute ischemic infarction, a brain MRI was then performed 95 minutes after the onset symptoms. It did not reveal significant acute infarction signals: no lesion was found on diffusion-weighted (DW) images (Figure 2), on apparent diffusion coefficient images, or on T2*-*weighted fluid-attenuated inversion recovery (FLAIR) images (Figure 3). The T2* images confirmed the absence of hemorrhage. This MRI was of inferior quality because of right artifacts due to osteosynthesis material for a right zygomatic fracture a few years previously. Cerebral arteriography was not performed, and the patient was admitted to the intensive care unit. Ionogram and complete blood count were normal. No drugs were found in the blood. The anticoagulant therapy was inefficient with an index normalized ratio of 1.2. The cerebrospinal fluid analysis was normal. Electroencephalogram recording did not show epileptiform activity.

**Figure 4:**
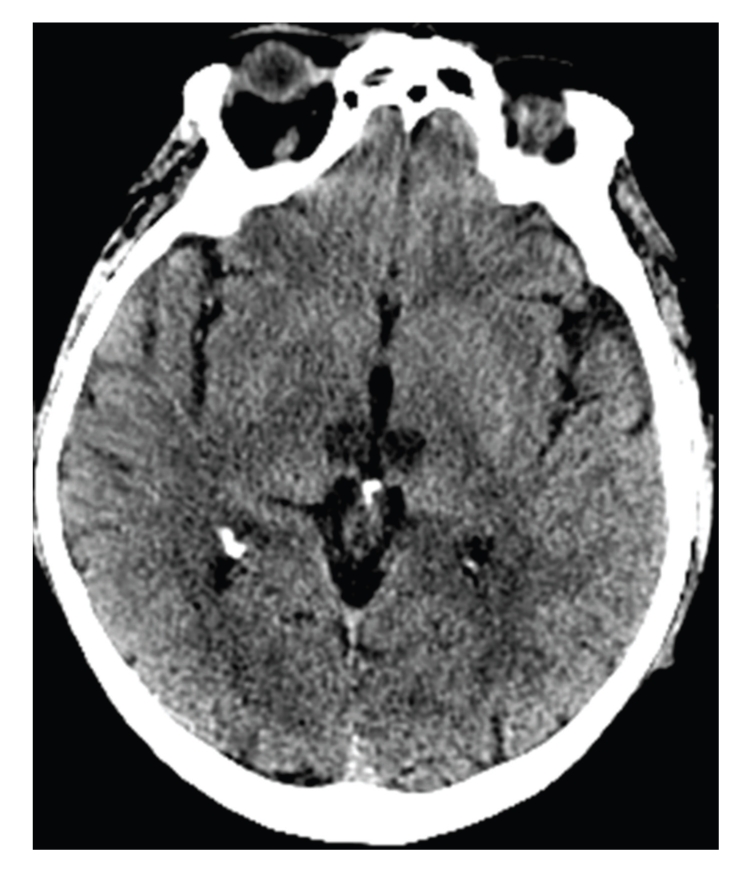
Head CT on day 2 showing bilateral symmetrical hypodensity of the medial part of the thalami corresponding to the occlusion of the artery of Percheron.

A new head CT was performed 48 hours later (Figure 4). It revealed a bilateral paramedian thalamic hypodensity corresponding to an obstruction of the artery of Percheron. A transesophageal echocardiography revealed a left atrial thrombus. The patient was anticoagulated with intravenous heparin. Sedation was stopped on day 3, and the patient was extubated on day 6. At first, neurologic examination showed altered levels of consciousness, eyes opening and vertical gaze palsy, and progressive response to simple orders. He was then gradually able to open his eyes normally, to speak, and to walk, but partial memory impairments were still present. After four weeks in a neurology department, the patient was discharged home in good condition with oral long-term anticoagulants.

## 3. Discussion

Bithalamic paramedian infarcts are rare, and it is painful to suspect them because of the complex anatomy causing large clinical variability [5–7]. The four main symptoms found in the literature are vertical gaze palsy (65%), memory impairment (58%), confusion (53%), and coma (42%) [7]. The prognosis is fairly good, as in our observation [4, 7, 8]. It may be ameliorated with the treatment of acute stroke, the thrombolytic therapy. It can be performed if the diagnosis of an acute stroke is done [9]. In stroke centers, MRI is the reference exam to diagnose acute brain ischemia. The combination of pathologic diffusion-weighted images and normal findings on T2-weighted FLAIR images in the paramedian thalami and possibly the mesencephalic area suggests an acute stroke of the artery of Percheron [9]. With a brain MRI showing an acute stroke, intravenous thrombolysis can be performed, if the deadline for achieving it is not exceeded (4 hours and 30 minutes after the onset symptoms). Presence of a hypersignal in T2-weighted FLAIR images and blood supply study also affect the decision of thrombolytic therapy. In our case, the early MRI was inconclusive and did not allow thrombolytic therapy. To our knowledge, this is the first case describing an artery of Percheron acute stroke with early nonsignificant MRI. 

Several reasons can explain the MRI results. The imaging was achieved with a head and neck antenna, which is probably less precise on this part of the brain compared with a head antenna. In addition, the brain MRI, performed 95 minutes after the onset of symptoms, was probably done earlier than in other reported cases. This demonstrates that modern neuroimaging is extremely valuable in making a positive diagnosis but normal imaging studies alone, however, do not exclude a vascular etiology. Small lesion size, brainstem lesions, small cortical lesions, and DW-imaging performed within a few hours after stroke onset are features associated with a particular risk of false-negative DW-images [10, 11]. In our case, there was no indication to perform a cerebral arteriography because of the negative MRI result. Furthermore, the efficiency and risks of such a therapy have not been evaluated. Moreover, such vessels are usually not visible on angiograms. Nevertheless, a case report describing successful artery of Percheron in situ thrombolysis in a patient similar to ours has been reported. However, the diagnosis suspicion was clinical with normal head CT, and MRI obtained after the arteriography [8].

## 4. Conclusion

In conclusion, our case indicates that a normal initial MRI cannot formally eliminate the diagnosis of acute stroke of the artery of Percheron, especially if performed very early or if the technical conditions are not optimal. These findings suggest the presentation of acute rostral brain stem stroke accompanied by a nonconclusive brain MRI should encourage to perform a new brain imaging by MRI within therapeutic times or should consider doing interventional explorations focused on the vertebrobasilar territory. On that basis, intravenous or in situ thrombolytic therapy should be performed. Interventional explorations focused on the vertebrobasilar territory have not been evaluated but may allow to confirm the diagnosis of Percheron artery lesion. However, only one successful case has been described. Anytime the initial imaging is normal, new brain imaging by MRI or CT scan should be performed within the first 48 hours.
